# Supplementary material for: Bone Formation in Zebrafish: The Significance of DAF-FM DA Staining for Nitric Oxide Detection
Source: Biomolecules. 2023 Dec 12;13(12):1780. doi: 10.3390/biom13121780 (PMC10742054; doi:10.3390/biom13121780)
Supplement: Supplementary file 1 [file biomolecules-13-01780-s001.zip › biomolecules-2709776-supplementary.pdf]

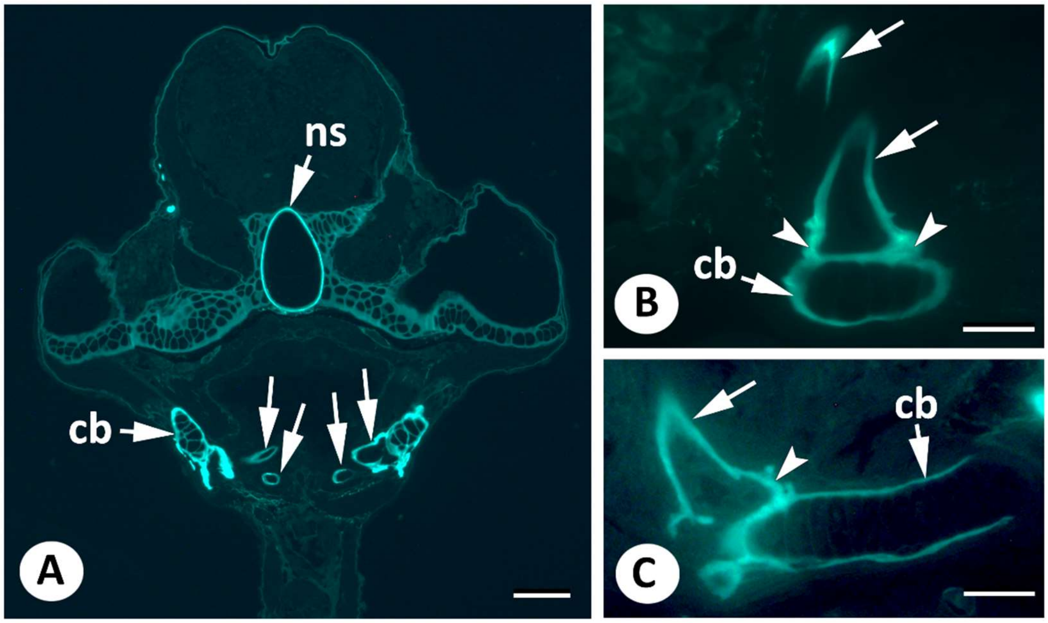

Figure S1. Semithin cross sections through 5dpf zebrafish live stained with DAF-FM DA showing labeling in the teeth.

A: overview section after 120 minutes of staining. B, C: magnifications of teeth after 20 minutes (B) and 3 hrs of staining (C). Teeth are indicated by arrows, the attachment pedicels by arrowheads. Note also staining of the bone around the tooth-bearing ceratobranchial 5 (cb) as well as of the notochord sheath (ns). Scale bar in A= 50 µm, in B & C= 20 µm.

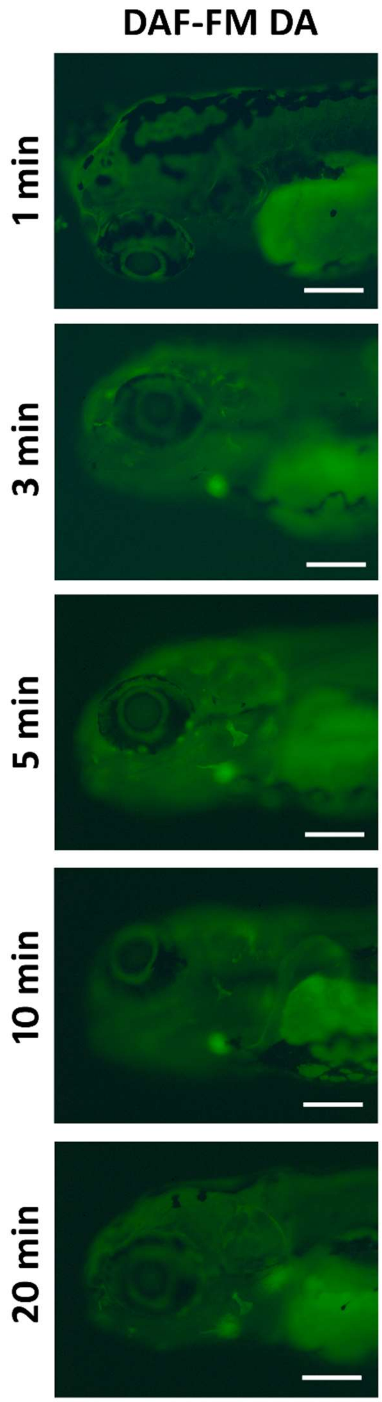

Figure S2. Live staining of 5 dpf zebrafish with DAF-FM DA for very short intervals.
Live imaging of the head region, at the time indicated on the left. The bulbus arteriosus is visible already after 3 minutes, the opercular bone after 5 minutes. Scale bars = 200 μm.

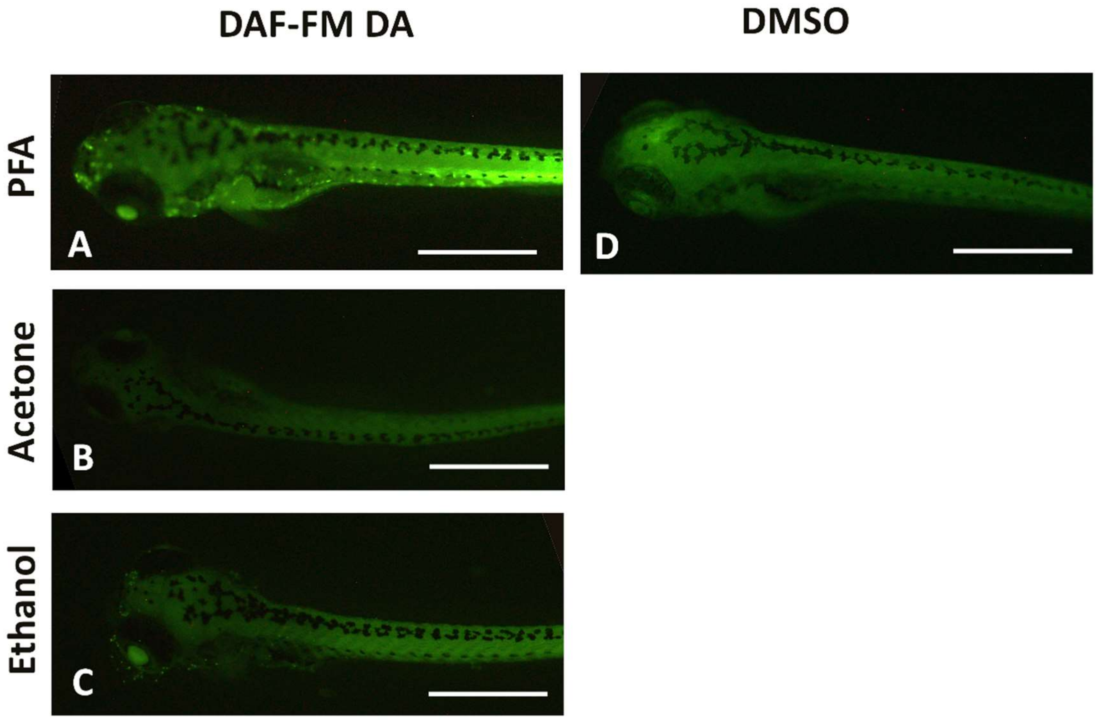

Figure S3. Staining with DAF-FM DA after fixation of 8 dpf zebrafish.
A-C: 3 hr staining with DAF-FM DA after fixation as indicated. D: 3 hr treatment of DMSO 0.1% after fixation in 4% PFA (compare with A). All images were taken strictly under the same illumination. Scale bars = 1 mm.
